# Discussion of some inequalities via fractional integrals

**DOI:** 10.1186/s13660-017-1609-3

**Published:** 2018-01-12

**Authors:** Mokhtar Kirane, Bessem Samet

**Affiliations:** 1 LaSIE, Faculté des Sciences et Technologies, Universié de La Rochelle, Avenue M. Crépeau, La Rochelle Cedex, 17042 France; 2 RUDN University, 6 Miklukho-Maklay St, Moscow, 117198 Russia; 3 Nonlinear Analysis and Applied Mathematics (NAAM) Research Group, Department of Mathematics, Faculty of Science, King Abdulaziz University, P.O. Box 80203, Jeddah, 21589 Saudi Arabia; 4 Department of Mathematics, College of Science, King Saud University, P.O. Box 2455, Riyadh, 11451 Saudi Arabia

**Keywords:** 26D10, 26A33, inequalities, fractional integrals

## Abstract

Recently, many generalizations and extensions of well-known inequalities were obtained via different kinds of fractional integrals. In this paper, we show that most of those results are particular cases of (or equivalent to) existing inequalities from the literature. As consequence, such results are not real generalizations.

## Introduction

Fractional calculus has received a great attention from many researchers in different disciplines. In particular, there has been an important interest in studying inequalities involving different kinds of fractional integrals. Unfortunately, as we will show later, most of the obtained results in this direction are particular cases of (or equivalent to) existing inequalities from the literature.

At first, let us recall briefly some definitions on fractional calculus that will be used later.

### Definition 1.1

(see [1])

Let $f\in L^{1}((a,b);\mathbb{R})$, $(a,b)\in\mathbb{R}^{2}$, $a< b$. The Riemann-Liouville fractional integrals $J_{a^{+}}^{\alpha}f$ and $J_{b^{-}}^{\alpha}f$ of order $\alpha>0$ are defined by

$$J_{a^{+}}^{\alpha}f(x)=\frac{1}{\Gamma(\alpha)} \int_{a}^{x}(x-t)^{\alpha -1}f(t)\,dt,\quad x>a, $$

and

$$J_{b^{-}}^{\alpha}f(x)=\frac{1}{\Gamma(\alpha)} \int_{x}^{b}(t-x)^{\alpha -1}f(t)\,dt,\quad x< b. $$

### Definition 1.2

(see [2])

Let $f\in L^{1}((a,b);\mathbb{R})$, $(a,b)\in\mathbb{R}^{2}$, $a< b$. The fractional integrals $I_{a}^{\alpha}f$ and $I_{b}^{\alpha}f$ of order $\alpha\in(0,1)$ are defined by

$$I_{a}^{\alpha}f(x)=\frac{1}{\alpha} \int_{a}^{x} \exp \biggl(-\frac{1-\alpha }{\alpha}(x-t) \biggr)f(t)\,dt,\quad x>a, $$

and

$$I_{b}^{\alpha}f(x)=\frac{1}{\alpha} \int_{x}^{b}\exp \biggl(-\frac{1-\alpha }{\alpha}(t-x) \biggr)f(t)\,dt,\quad x< b. $$

The paper is organized as follows. Section 2 is devoted to results and discussions. More precisely, in Section 2.1, we discuss some recent Hermite-Hadamard-type inequalities via different kinds of fractional integrals. We show that such inequalities are particular cases of (or equivalent to) Fejér inequality. In Section 2.2, we discuss a Gruss-type inequality involving fractional integrals, which was obtained by Dahmani *et al*. [3]. We show that such inequality is a particular case of a weighted version of Gruss inequality, which was established by Dragomir [4]. In Section 2.3, we discuss a fractional-type inequality related to weighted Chebyshev’s functional, which was presented by Dahmani [5]. We show that such an inequality is not new, and it is equivalent to an existing inequality proved by Dragomir [4]. We end the paper with a conclusion in Section 3.

## Results and discussions

In this section, we discuss several recent inequalities involving different types of fractional integrals, and we prove that these inequalities are particular cases of (or equivalent to) previous existing results from the literature.

### On Hermite-Hadamard-type inequalities involving fractional integrals

Let $f: [a,b]\to\mathbb{R}$, $(a,b)\in\mathbb{R}^{2}$, $a< b$, be a convex function. Then (see [6–8])

1 $$ f \biggl(\frac{a+b}{2} \biggr)\leq\frac{1}{b-a} \int_{a}^{b} f(t)\,dt\leq \frac{f(a)+f(b)}{2}. $$

Inequality (1) was known in the literature as Hermite-Hadamard inequality.

In [9], Fejér established the following result, which contains a weighted generalization of (1).

#### Theorem 2.1

*Let* $f: [a,b]\to\mathbb{R}$, $(a,b)\in\mathbb{R}^{2}$, $a< b$, *be a convex function*. *Let* $w\in L^{1}((a,b);\mathbb{R})$ *be non*-*negative and symmetric to* $\frac{a+b}{2}$. *Then*

2 $$ f \biggl(\frac{a+b}{2} \biggr) \int_{a}^{b} w(t)\,dt \leq \int_{a}^{b} f(t)w(t)\, dt\leq\frac{f(a)+f(b)}{2} \int_{a}^{b} w(t)\,dt. $$

Observe that (1) follows from (2) by taking $w\equiv1$.

Recently, many generalizations and extensions of (1) were derived by many authors using fractional integrals. In this direction, we refer the reader to [2, 10–14], and the references therein. In this section, we show that most of those results are particular cases of (or equivalent to) Theorem 2.1. To simplify the presentation, we will consider only the results obtained in [2, 12, 14].

In [14], Sarikaya *et al*. established the following Hermite-Hadamard-type inequality via Riemann-Liouville integrals.

#### Theorem 2.2

*Let* $f\in L^{1}([a,b];\mathbb{R})$, $(a,b)\in\mathbb{R}^{2}$, $a< b$, *be a convex function*. *Then*

3 $$ f \biggl(\frac{a+b}{2} \biggr)\leq\frac{\Gamma(\alpha+1)}{2(b-a)^{\alpha}} \bigl[J_{a^{+}}^{\alpha}f(b)+J_{b^{-}}^{\alpha}f(a) \bigr]\leq\frac{f(a)+f(b)}{2}, $$

*where* $\alpha>0$.

Note that in [14], it is supposed that $a\geq0$ and *f* is a non-negative function. We will show later that such assumptions are superfluous.

In [12], Işcan presented the following result.

#### Theorem 2.3

*Let* $f\in L^{1}([a,b];\mathbb{R})$, $(a,b)\in\mathbb{R}^{2}$, $a< b$, *be a convex function*. *Let* $g\in L^{1}((a,b);\mathbb{R})$ *be non*-*negative and symmetric to* $\frac{a+b}{2}$. *Then*

4 $$ \begin{aligned}[b] f \biggl(\frac{a+b}{2} \biggr) \bigl[J_{a^{+}}^{\alpha}g(b)+J_{b^{-}}^{\alpha}g(a) \bigr]&\leq \bigl[J_{a^{+}}^{\alpha}(fg) (b)+J_{b^{-}}^{\alpha}(fg) (a) \bigr]\\&\leq\frac{f(a)+f(b)}{2} \bigl[J_{a^{+}}^{\alpha}g(b)+J_{b^{-}}^{\alpha}g(a) \bigr],\end{aligned} $$

*where* $\alpha>0$.

In [2], Kirane and Torebek presented the following result.

#### Theorem 2.4

*Let* $f\in L^{1}([a,b];\mathbb{R})$, $(a,b)\in\mathbb{R}^{2}$, $a< b$, *be a convex function*. *Then*

5 $$ f \biggl(\frac{a+b}{2} \biggr)\leq \frac{1-\alpha}{2(1-\exp(-\mathcal {A}))} \bigl[I_{a}^{\alpha}f(b)+I_{b}^{\alpha}f(a) \bigr]\leq\frac{f(a)+f(b)}{2}, $$

*where* $\alpha\in(0,1)$ *and* $\mathcal{A}=\frac{1-\alpha}{\alpha}(b-a)$.

In [2], it is supposed that $a\geq0$ and *f* is a non-negative function. We will show later that such assumptions are superfluous.

Our first observation is formulated by the following theorem.

#### Theorem 2.5

*Theorem* 2.1 ⇒ *Theorem* 2.2.

#### Proof

Let us suppose that all assumptions of Theorem 2.2 are satisfied. Let us define the function *w* by

$$w(t)=\frac{1}{\Gamma(\alpha)} \bigl((b-t)^{\alpha-1}+(t-a)^{\alpha -1} \bigr), \quad a< t< b. $$

Clearly, $w \in L^{1}((a,b);\mathbb{R})$, and it is a non-negative function. Moreover, for all $t\in(a,b)$, we have

$$\begin{aligned} \Gamma(\alpha)w(a+b-t)&=\bigl(b-(a+b-t)\bigr)^{\alpha-1}+\bigl((a+b-t)-a \bigr)^{\alpha -1}\\&=(t-a)^{\alpha-1}+(b-t)^{\alpha-1}=\Gamma(\alpha)w(t).\end{aligned} $$

Therefore, *w* is symmetric to $\frac{a+b}{2}$. Now, by Theorem 2.1, it follows from (2) that

6 $$ f \biggl(\frac{a+b}{2} \biggr) \int_{a}^{b} w(t)\,dt \leq \int_{a}^{b} f(t)w(t)\, dt\leq\frac{f(a)+f(b)}{2} \int_{a}^{b} w(t)\,dt. $$

On the other hand, we have

7 $$ \Gamma(\alpha) \int_{a}^{b} w(t)\,dt= \int_{a}^{b} (b-t)^{\alpha-1} \, dt+ \int_{a}^{b} (t-a)^{\alpha-1} \,dt = \frac{2}{\alpha} (b-a)^{\alpha}. $$

Moreover, we have

8 $$ \begin{aligned}[b] \int_{a}^{b} f(t)w(t)\,dt&=\frac{1}{\Gamma(\alpha)} \int_{a}^{b} (b-t)^{\alpha-1} f(t)\,dt+ \frac{1}{\Gamma(\alpha)} \int_{a}^{b} (t-a)^{\alpha-1} f(t)\,dt \\ &=J_{a^{+}}^{\alpha}f(b)+J_{b^{-}}^{\alpha}f(a).\end{aligned} $$

Therefore, using (6), (7), and (8), we obtain

$$f \biggl(\frac{a+b}{2} \biggr)\leq\frac{\Gamma(\alpha+1)}{2(b-a)^{\alpha}} \bigl[J_{a^{+}}^{\alpha}f(b)+J_{b^{-}}^{\alpha}f(a) \bigr]\leq\frac{f(a)+f(b)}{2}, $$

which is inequality (3). Therefore, we proved that Theorem 2.1 ⇒ Theorem 2.2. □

Next, we have the following observation concerning Theorem 2.3.

#### Theorem 2.6

*Theorem* 2.1 ⇔ *Theorem* 2.3.

#### Proof

Let us suppose that all assumptions of Theorem 2.3 are satisfied. Let us define the function *w* by

$$w(t)=\frac{g(t)}{\Gamma(\alpha)} \bigl((b-t)^{\alpha-1}+(t-a)^{\alpha -1} \bigr), \quad a< t< b. $$

Clearly, $w \in L^{1}((a,b);\mathbb{R})$, and it is non-negative and symmetric to $\frac{a+b}{2}$ (since *g* is symmetric to $\frac {a+b}{2}$). By Theorem 2.1, it follows from (2) that

9 $$ f \biggl(\frac{a+b}{2} \biggr) \int_{a}^{b} w(t)\,dt \leq \int_{a}^{b} f(t)w(t)\, dt\leq\frac{f(a)+f(b)}{2} \int_{a}^{b} w(t)\,dt. $$

On the other hand, we have

10 $$ \begin{aligned}[b] \int_{a}^{b} w(t)\,dt&=\frac{1}{\Gamma(\alpha)} \int_{a}^{b} g(t) \bigl((b-t)^{\alpha-1}+(t-a)^{\alpha-1} \bigr)\,dt \\ &=\frac{1}{\Gamma(\alpha)} \int_{a}^{b} (b-t)^{\alpha-1} g(t)\, dt+ \frac{1}{\Gamma(\alpha)} \int_{a}^{b} (t-a)^{\alpha-1} g(t)\,dt \\ &= J_{a^{+}}^{\alpha}g(b)+J_{b^{-}}^{\alpha}g(a).\end{aligned} $$

Moreover,

11 $$ \begin{aligned}[b] \int_{a}^{b} f(t)w(t)\,dt&= \frac{1}{\Gamma(\alpha)} \int_{a}^{b} f(t)g(t) \bigl((b-t)^{\alpha-1}+(t-a)^{\alpha-1} \bigr)\,dt \\ &=\frac{1}{\Gamma(\alpha)} \int_{a}^{b} (b-t)^{\alpha-1} f(t)g(t)\,dt+ \frac{1}{\Gamma(\alpha)} \int_{a}^{b} (t-a)^{\alpha-1} f(t)g(t)\,dt \\ &= J_{a^{+}}^{\alpha}(fg) (b)+J_{b^{-}}^{\alpha}(fg) (a).\end{aligned} $$

Combining (9), (10), and (11), we obtain

$$f \biggl(\frac{a+b}{2} \biggr) \bigl[J_{a^{+}}^{\alpha}g(b)+J_{b^{-}}^{\alpha}g(a) \bigr]\leq \bigl[J_{a^{+}}^{\alpha}(fg) (b)+J_{b^{-}}^{\alpha}(fg) (a) \bigr]\leq\frac{f(a)+f(b)}{2} \bigl[J_{a^{+}}^{\alpha}g(b)+J_{b^{-}}^{\alpha}g(a) \bigr], $$

which is inequality (4). Therefore, we proved that Theorem 2.1 ⇒ Theorem 2.3.

Now, suppose that all the assumptions of Theorem 2.1 are satisfied. Taking $g=w$ and $\alpha=1$ in (4), we obtain (2). Therefore, we proved that Theorem 2.3 ⇒ Theorem 2.1. □

Our comment on Theorem 2.4 is formulated by the following theorem.

#### Theorem 2.7

*Theorem* 2.1 ⇒ *Theorem* 2.4.

#### Proof

Let us suppose that all assumptions of Theorem 2.4 are satisfied. Let us define the function *w* by

$$w(t)=\frac{1}{\alpha} \biggl(\exp \biggl(-\frac{1-\alpha}{\alpha }(b-t) \biggr)+\exp \biggl(-\frac{1-\alpha}{\alpha}(t-a) \biggr) \biggr),\quad a\leq t\leq b. $$

It can be easily seen that $w \in L^{1}((a,b);\mathbb{R})$, and it is non-negative and symmetric to $\frac{a+b}{2}$. By Theorem 2.1, it follows from (2) that

12 $$ f \biggl(\frac{a+b}{2} \biggr) \int_{a}^{b} w(t)\,dt \leq \int_{a}^{b} f(t)w(t)\, dt\leq\frac{f(a)+f(b)}{2} \int_{a}^{b} w(t)\,dt. $$

On the other hand, we have

13 $$ \begin{aligned}[b] \int_{a}^{b} w(t)\,dt&=\frac{1}{\alpha} \int_{a}^{b} \biggl(\exp \biggl(- \frac {1-\alpha}{\alpha}(b-t) \biggr)+\exp \biggl(-\frac{1-\alpha}{\alpha }(t-a) \biggr) \biggr)\,dt \\ &=\frac{1}{\alpha} \int_{a}^{b} \exp \biggl(-\frac{1-\alpha}{\alpha }(b-t) \biggr)\,dt+\frac{1}{\alpha} \int_{a}^{b} \exp \biggl(-\frac{1-\alpha }{\alpha}(t-a) \biggr)\,dt \\ &= \frac{1}{1-\alpha} \biggl(1-\exp \biggl(-\frac{1-\alpha}{\alpha }(b-a) \biggr) \biggr)-\frac{1}{1-\alpha} \biggl(\exp \biggl(-\frac{1-\alpha }{\alpha}(b-a) \biggr)-1 \biggr) \\ &= \frac{2}{1-\alpha} \biggl(1-\exp \biggl(-\frac{1-\alpha}{\alpha }(b-a) \biggr) \biggr) \\ &=\frac{2}{1-\alpha} \bigl(1-\exp (-\mathcal{A} ) \bigr).\end{aligned} $$

Moreover, we have

14 $$ \begin{aligned}[b] \int_{a}^{b} f(t)w(t)\,dt&=\frac{1}{\alpha} \int_{a}^{b} \biggl(\exp \biggl(- \frac{1-\alpha}{\alpha}(b-t) \biggr)+\exp \biggl(-\frac{1-\alpha}{\alpha }(t-a) \biggr) \biggr)f(t)\,dt \\ &=\frac{1}{\alpha} \int_{a}^{b} \exp \biggl(-\frac{1-\alpha}{\alpha }(b-t) \biggr)f(t)\,dt+\frac{1}{\alpha} \int_{a}^{b} \exp \biggl(-\frac{1-\alpha }{\alpha}(t-a) \biggr)f(t)\,dt \\ &=I_{a}^{\alpha}f(b)+I_{b}^{\alpha}f(a).\end{aligned} $$

Combining (12), (13), and (14), we obtain

$$f \biggl(\frac{a+b}{2} \biggr)\leq \frac{1-\alpha}{2(1-\exp(-\mathcal {A}))} \bigl[I_{a}^{\alpha}f(b)+I_{b}^{\alpha}f(a) \bigr]\leq\frac{f(a)+f(b)}{2}, $$

which is inequality (5). Therefore, we proved that Theorem 2.1 ⇒ Theorem 2.4. □

### Discussion of Gruss-type inequalities involving fractional integrals

In 1935, Gruss [15] proved the following result.

#### Theorem 2.8

*Let* $f,g\in L^{1}((a,b);\mathbb{R})$, $(a,b)\in\mathbb{R}^{2}$, $a< b$. *Suppose that there exist constants* $m,M,p,P\in\mathbb{R}$ *such that*

$$m\leq f(x)\leq M,\qquad p\leq g(x)\leq P,\quad a\leq x\leq b. $$

*Then*

15 $$ \begin{aligned}[b] &\biggl\vert \frac{1}{b-a} \int_{a}^{b} f(x)g(x)\,dx- \biggl(\frac{1}{b-a} \int_{a}^{b} f(x)\,dx \biggr) \biggl( \frac{1}{b-a} \int_{a}^{b} g(x)\,dx \biggr) \biggr\vert \\&\quad\leq \frac{(M-m)(P-p)}{4}.\end{aligned} $$

Inequality (15) has evoked the interest of many researchers, and several generalizations of this inequality have appeared in the literature. In particular, in 1998, Dragomir [4] established the following interesting generalization, which provides a weighted version of the Gruss inequality.

#### Theorem 2.9

*Let* $f,g\in L^{1}((a,b);\mathbb{R})$, $(a,b)\in\mathbb{R}^{2}$, $a< b$. *Suppose that there exist constants* $m,M,p,P\in\mathbb{R}$ *such that*

$$m\leq f(x)\leq M,\qquad p\leq g(x)\leq P,\quad a\leq x\leq b. $$

*Let* $h\in L^{1}((a,b);\mathbb{R})$ *be a non*-*negative function such that* $\int_{a}^{b} h(x)\,dx>0$. *Then*

16 $$ \begin{aligned}[b]&\biggl\vert \int_{a}^{b} h(x)\,dx \int_{a}^{b} f(x)g(x)h(x)\,dx- \int_{a}^{b} f(x)h(x)\,dx \int_{a}^{b} g(x)h(x)\,dx \biggr\vert \\&\quad \leq \frac{(M-m)(P-p)}{4} \biggl( \int_{a}^{b} h(x)\,dx \biggr)^{2}.\end{aligned} $$

Observe that (15) follows from (16) by taking $h\equiv1$.

After the publication of reference [4], in 2010, Dahmani and Tabharit [3] presented the following result.

#### Theorem 2.10

*Let* *f* *and* *g* *be two integrable functions on* $[0,\infty)$. *Suppose that there exist constants* $m,M,p,P\in\mathbb{R}$ *such that*

$$m\leq f(x)\leq M,\qquad p\leq g(x)\leq P,\quad x\geq0. $$

*Let* $p\in L^{1}([0,\infty);\mathbb{R})$ *be a non*-*negative function such that* $J_{0^{+}}^{\alpha}p(T)>0$, *for all* $T>0$. *Then*

17 $$ \bigl\vert J_{0^{+}}^{\alpha}p (T)J_{0^{+}}^{\alpha}(pfg) (T)-J_{0^{+}}^{\alpha}(pf) (T)J_{0^{+}}^{\alpha}(pg) (T) \bigr\vert \leq \bigl(J_{0^{+}}^{\alpha}p(T) \bigr)^{2} \frac{(M-m)(P-p)}{4} $$

*for all* $\alpha>0$ *and* $T>0$.

We have the following observation.

#### Theorem 2.11

*Theorem* 2.9 ⇒ *Theorem* 2.10.

#### Proof

Suppose that all assumptions of Theorem 2.10 are satisfied. Let us fix $\alpha>0$ and $T>0$. Let

$$h(x)=\frac{1}{\Gamma(\alpha)}(T-x)^{\alpha-1} p(x) ,\quad0\leq x< T. $$

Clearly, *h* is a non-negative function. Moreover, we have

18 $$ \int_{0}^{T} h(x)\,dx\geq\frac{1}{\Gamma(\alpha)} \int_{0}^{T} (T-x)^{\alpha -1} p(x)\,dx =J_{0^{+}}^{\alpha}p(T)>0. $$

Therefore, using (18), by Theorem 2.9 with $(a,b)=(0,T)$, it follows from (16) that

19 $$ \begin{aligned}[b] &\biggl\vert J_{0^{+}}^{\alpha}p(T) \int_{0}^{T} f(x)g(x)h(x)\,dx- \int_{0}^{T} f(x)h(x)\, dx \int_{0}^{T} g(x)h(x)\,dx \biggr\vert \\&\quad \leq \frac{(M-m)(P-p)}{4} \bigl(J_{0^{+}}^{\alpha}p(T) \bigr)^{2}.\end{aligned} $$

On the other hand, observe that

20 $$\begin{aligned}& \int_{0}^{T} f(x)g(x)h(x)\,dx = J_{0^{+}}^{\alpha}(pfg) (T), \end{aligned}$$

21 $$\begin{aligned}& \int_{0}^{T} f(x)h(x)\,dx = J_{0^{+}}^{\alpha}(pf) (T), \end{aligned}$$

22 $$\begin{aligned}& \int_{0}^{T} g(x)h(x)\,dx = J_{0^{+}}^{\alpha}(pg) (T). \end{aligned}$$

Combining (19), (20), (21), and (22), we obtain inequality (17). Therefore, we proved that Theorem 2.9⇒ Theorem 2.10. □

### Discussion of fractional-type inequalities related to the weighted Chebyshev’s functional

Let us introduce Chebyshev functional

$$M(f,g,p)= \int_{0}^{T} p(x)\,dx \int_{0}^{T}p(x)f(x)g(x)\,dx- \int_{0}^{T}p(x)f(x)\, dx \int_{0}^{T}p(x)g(x)\,dx, $$

where $T>0$, *f* and *g* are two integrable functions on $[0,T]$, and *p* is a non-negative and integrable function on $[0,T]$.

In [4], Dragomir proved the following interesting result.

#### Theorem 2.12

*Suppose that* *f* *and* *g* *are two differentiable functions*, $f', g'\in L^{\infty}((0,T);\mathbb{R})$, *and* *p* *is a non*-*negative and integrable function on* $[0,T]$. *Then*

$$\big|M(f,g,p)\big|\leq\big\| f'\big\| _{\infty}\big\| g' \big\| _{\infty}\biggl( \int_{0}^{T} p(x)\,dx \int _{0}^{T}x^{2}p(x)\,dx- \biggl( \int_{0}^{T}xp(x)\,dx \biggr)^{2} \biggr). $$

Observe that if we assume also that *f* and *g* have the same monotony, then

$$M(f,g,p)\geq0. $$

Indeed, in this case, we have

$$F(x,y):=\frac{(f(x)-f(y))(g(x)-g(y))}{2}\geq0,\quad(x,y)\in (0,T)\times(0,T). $$

Therefore,

$$\int_{0}^{T} \int_{0}^{T} p(x)p(y)F(x,y)\,dx\,dy\geq0. $$

On the other hand, it can be easily seen that

$$M(f,g,p)= \int_{0}^{T} \int_{0}^{T} p(x)p(y)F(x,y)\,dx\,dy\geq0. $$

Then we can state the following result.

#### Theorem 2.13

*Suppose that* *f* *and* *g* *are two differentiable functions having the same monotony*, $f', g'\in L^{\infty}((0,T);\mathbb{R})$, *and* *p* *is a non*-*negative and integrable function on* $[0,T]$. *Then*

23 $$ 0\leq M(f,g,p)\leq\big\| f'\big\| _{\infty}\big\| g'\big\| _{\infty}\biggl( \int_{0}^{T} p(x)\,dx \int _{0}^{T}x^{2}p(x)\,dx- \biggl( \int_{0}^{T}xp(x)\,dx \biggr)^{2} \biggr). $$

In [5], Dahmani presented the following fractional version of Theorem 2.13.

#### Theorem 2.14

*Let* *p* *be a non*-*negative function on* $[0,\infty)$ *and let* *f* *and* *g* *be two differentiable functions having the same monotony on* $[0,\infty )$. *If* $f', g'\in L^{\infty}((0,\infty);\mathbb{R})$, *then*

24 $$ \begin{aligned}[b]0&\leq J_{0^{+}}^{\alpha}p(T)J_{0^{+}}^{\alpha}(pfg) (T)- J_{0^{+}}^{\alpha}(pf) (T)J_{0^{+}}^{\alpha}(pg) (T)\\ &\leq\big\| f'\big\| _{\infty}\big\| g'\big\| _{\infty}\bigl(J_{0^{+}}^{\alpha}p(T)J_{0^{+}}^{\alpha}\bigl(T^{2}p\bigr) (T)-\bigl(J_{0^{+}}^{\alpha}(Tp) (T) \bigr)^{2} \bigr),\end{aligned} $$

*where* $\alpha>0$.

We have the following observation concerning Theorem 2.14.

#### Theorem 2.15

*Theorem* 2.13 ⇔ *Theorem* 2.14.

#### Proof

Suppose that all assumptions of Theorem 2.14 are satisfied. Let us introduce the function

$$\widetilde{p}(x)=(T-x)^{\alpha-1} p(x),\quad0\leq x< T. $$

Clearly, *p̃* is non-negative and

25 $$ \int_{0}^{T} \widetilde{p}(x)\,dx=J_{0^{+}}^{\alpha}p(T). $$

By Theorem 2.13, it follows from (23) that

26 $$ 0\leq M(f,g,\widetilde{p})\leq\big\| f'\big\| _{\infty}\big\| g'\big\| _{\infty}\biggl( \int_{0}^{T} \widetilde{p}(x)\,dx \int_{0}^{T}x^{2}\widetilde{p}(x)\,dx- \biggl( \int _{0}^{T}x\widetilde{p}(x)\,dx \biggr)^{2} \biggr). $$

On the other hand, it can be easily seen that

27 $$\begin{aligned}& \int_{0}^{T} \widetilde{P}(x)f(x)g(x)\,dx= J_{0^{+}}^{\alpha}(pfg) (T), \end{aligned}$$

28 $$\begin{aligned}& \int_{0}^{T} \widetilde{P}(x)f(x)\,dx= J_{0^{+}}^{\alpha}(pf) (T), \end{aligned}$$

29 $$\begin{aligned}& \int_{0}^{T} \widetilde{P}(x)g(x)\,dx= J_{0^{+}}^{\alpha}(pg) (T), \end{aligned}$$

30 $$\begin{aligned}& \int_{0}^{T} x^{2}\widetilde{P}(x)\,dx= J_{0^{+}}^{\alpha}\bigl(T^{2}p\bigr) (T), \end{aligned}$$

31 $$\begin{aligned}& \int_{0}^{T} x\widetilde{P}(x)\,dx= J_{0^{+}}^{\alpha}(Tp) (T). \end{aligned}$$

Using (25), (27), (28), and (29), we obtain

32 $$ M(f,g,\widetilde{p})=J_{0^{+}}^{\alpha}p(T)J_{0^{+}}^{\alpha}(pfg) (T)- J_{0^{+}}^{\alpha}(pf) (T)J_{0^{+}}^{\alpha}(pg) (T). $$

Next, using (26), (25), (30), and (31), we obtain

$$\begin{aligned} 0&\leq J_{0^{+}}^{\alpha}p(T)J_{0^{+}}^{\alpha}(pfg) (T)- J_{0^{+}}^{\alpha}(pf) (T)J_{0^{+}}^{\alpha}(pg) (T)\\ &\leq\big\| f'\big\| _{\infty}\big\| g'\big\| _{\infty}\bigl(J_{0^{+}}^{\alpha}p(T)J_{0^{+}}^{\alpha}\bigl(T^{2}p\bigr) (T)-\bigl(J_{0^{+}}^{\alpha}(Tp) (T) \bigr)^{2} \bigr),\end{aligned} $$

which is inequality (24). Therefore, we proved that Theorem 2.13 ⇒ Theorem 2.14.

Finally, taking $\alpha=1$ in Theorem 2.14, we obtain the result given by Theorem 2.13. Therefore, we have Theorem 2.14 ⇒ Theorem 2.13. □

## Conclusion

Recently, a lot of papers are published concerning inequalities involving different kinds of fractional integrals. In this paper, we proved that most of those inequalities are just particular cases of (or equivalent to) existing results form the literature. We discussed only three types of inequalities: Hermite-Hadamard- type inequalities, Gruss-type inequalities, and an inequality related to Chebyshev’s functional. However, the used technique can be also applied for many other published results.
